# Feasibility of Diffusion Tractography for the Reconstruction of Intra-Thalamic and Cerebello-Thalamic Targets for Functional Neurosurgery: A Multi-Vendor Pilot Study in Four Subjects

**DOI:** 10.3389/fnana.2016.00076

**Published:** 2016-07-12

**Authors:** András Jakab, Beat Werner, Marco Piccirelli, Kázmér Kovács, Ernst Martin, John S. Thornton, Tarek Yousry, Gabor Szekely, Ruth O‘Gorman Tuura

**Affiliations:** ^1^Center for Magnetic Resonance Imaging Research, University Children’s HospitalZürich, Switzerland; ^2^Computational Imaging Research Lab, Department of Biomedical Imaging and Image-Guided Therapy, Medical University of ViennaVienna, Austria; ^3^Department of Neuroradiology, University Hospital ZürichZürich, Switzerland; ^4^Department of Biomedical Imaging and Laboratory Science, University of DebrecenDebrecen, Hungary; ^5^University College London Institute of NeurologyLondon, UK; ^6^Computer Vision Laboratory, ETH ZürichZürich, Switzerland

**Keywords:** diffusion MRI, diffusion tensor imaging, brain connectivity, cerebello-thalamic tract, functional neurosurgery

## Abstract

Functional stereotactic neurosurgery by means of deep brain stimulation or ablation provides an effective treatment for movement disorders, but the outcome of surgical interventions depends on the accuracy by which the target structures are reached. The purpose of this pilot study was to evaluate the feasibility of diffusion tensor imaging (DTI) based probabilistic tractography of deep brain structures that are commonly used for pre- and perioperative targeting for functional neurosurgery. Three targets were reconstructed based on their significance as intervention sites or as a no-go area to avoid adverse side effects: the connections propagating from the thalamus to (1) primary and supplementary motor areas, (2) to somatosensory areas and the cerebello-thalamic tract (CTT). We evaluated the overlap of the reconstructed connectivity based targets with corresponding atlas based data, and tested the inter-subject and inter-scanner variability by acquiring repeated DTI from four volunteers, and on three MRI scanners with similar sequence parameters. Compared to a 3D histological atlas of the human thalamus, moderate overlaps of 35-50% were measured between connectivity- and atlas based volumes, while the minimal distance between the centerpoints of atlas and connectivity targets was 2.5 mm. The variability caused by the MRI scanner was similar to the inter-subject variability, except for connections with the postcentral gyrus where it was higher. While CTT resolved the anatomically correct trajectory of the tract individually, high volumetric variability was found across subjects and between scanners. DTI can be applied in the clinical, preoperative setting to reconstruct the CTT and to localize subdivisions within the lateral thalamus. In our pilot study, such subdivisions moderately matched the borders of the ventrolateral-posteroventral (VL_pv_) nucleus and the ventral-posterolateral (VPL) nucleus. Limitations of the currently used standard DTI protocols were exacerbated by large scanner-to-scanner variability of the connectivity-based targets.

## Introduction

Functional stereotactic neurosurgery by means of deep brain stimulation or ablation provides an effective treatment for movement disorders including Parkinson’s disease (Benabid et al., 1996; Ohye, 2009), essential tremor and dystonia (Baizabal-Carvallo et al., 2014). Since the brain and in particular the thalamic and subthalamic regions frequently targeted for functional stereotactic neurosurgery are tightly packed with neuronal circuits, the outcome of surgical interventions depends critically on the accuracy by which the target structures are reached (Hariz, 2002; Lanotte et al., 2002). Errors in targeting can compromise the efficacy of the treatment and lead to adverse side effects such as motor deficits (Baizabal-Carvallo and Jankovic, 2016), hypomania (Chopra et al., 2012), impulse control disorders (Amami et al., 2015), hemi-neglect, and dysarthria (Tripoliti et al., 2011). Therefore, novel and improved targeting approaches are required to provide optimal image guidance for tailored and personalized therapy.

Localization of the target structures can be performed either directly from MRI or indirectly from atlas coordinates and predefined anatomical landmarks (Lemaire et al., 2007). Given the inter-subject variability in anatomical position of the target nuclei (Morel et al., 1997), direct targeting is arguably more accurate, but this approach is limited by the low contrast and relatively low visibility of the target structures on MRI. This low visibility represents a particular challenge for neurosurgical targeting in the thalamus, which contains important target structures for stereotactic surgical interventions by means of ablative therapies such as centrolateral thalamotomy for pain, or deep-brain stimulation of the nucleus Vim (Benabid et al., 1996; Gallay et al., 2008; Martin et al., 2009).

In the clinical setting, while the relaxation properties depicted by T1- or T2-weighted MRI offer limited insight into the anatomy of the thalamus, recently, diffusion MRI based techniques have been suggested as a way to visualize various subdivisions representing possible target locations within the thalamus (Behrens et al., 2003a; Wiegell et al., 2003; Johansen-Berg et al., 2005; Traynor et al., 2010). Diffusion MRI and DTI can be used to estimate the intra-voxel incoherent diffusion driven motion of water molecules (Le Bihan and Johansen-Berg, 2012). The local orientation of diffusion correlates with the direction of axonal fibers within an image voxel, providing the basis for various tractography techniques that enable the macroscopic reconstruction of white matter structures, as well as the connectional anatomy of subcortical structures (Jonasson et al., 2007; Zhang et al., 2010; O’Muircheartaigh et al., 2011). While the most common way of using tractography is done by charting the white matter trajectories emerging from a “seed” area, the back-propagation of remote connections to a seed region allows the parcellation of cortical and subcortical areas based on their in- and outgoing connectivity (Behrens et al., 2003a; Johansen-Berg et al., 2004; Draganski et al., 2008). Furthermore, tractography can be used to directly reconstruct fiber pathways emerging from the thalamus, as potential white matter targets for functional neurosurgery (Kwon et al., 2011).

The direct mapping of the thalamic nuclei, such as the Vim through diffusion tractography is not possible, since the lateral thalamus acts as a relay station with specific and somatotopically organized cortico-thalamic connections (Kievit and Kuypers, 1977). However, the subdivision of the thalamus based on its remote connectivity is possible (connectivity-based segmentation: Johansen-Berg et al., 2005). Thalamic mapping utilizing various tractography and connectivity-based segmentation techniques has been suggested for preoperative prediction of target location for functional neurosurgery (Pouratian et al., 2011; Hyam et al., 2012; Lujan et al., 2012; Hunsche et al., 2013; Sudhyadhom et al., 2013; Anthofer et al., 2014; Torres et al., 2014; Avecillas-Chasin et al., 2016). Some previous studies have used connectivity-based segmentation methods to localize the intra-thalamic representation of motor and premotor connections to find the most probable location of the Vim target for functional neurosurgery (Pouratian et al., 2011; Hyam et al., 2012), while other studies investigated the link between the outcome of neurosurgery and the anatomical configuration of adjacent fiber tracts predicted by tractography (Butson et al., 2006; Anthofer et al., 2014). However, connectivity-based methods can also localize regions adjacent to the possible target areas with strong somatosensory connections, such as connections to the postcentral gyrus, providing a means to define safety margins around possible target areas to avoid adverse treatment effects. The definition of such “no-go” areas is particularly important for approaches such as MRI-guided high intensity focused ultrasound ablation, which relies heavily on the preoperative definition of possible target sites, as the intraoperative monitoring of adverse clinical symptoms is limited without intraoperative physiological monitoring.

In addition to the thalamic nuclei, other structures including the dentato-rubro-thalamic or CTT encompass fibers that act to coordinate the initiation, planning and timing of movement, and accordingly have also been proposed as a putative target for the alleviation of symptoms in various motor disorders (Ohye et al., 1993; Gallay et al., 2008; Schlaier et al., 2015). Diffusion tractography has been used successfully to reconstruct this structure in the clinical setting (Coenen et al., 2011a; Kwon et al., 2011; Schlaier et al., 2015), and has also been used to correlate outcome with the adjacency of this tract. However, studies investigating the link between outcome and the adjacency of the CTT have reported mixed accuracy, calling for a more precise evaluation of the reproducibility of the localization of this tract with tractography (Schlaier et al., 2015). A further challenge is to evaluate the correspondence of the tractography based target sites to the intra-operatively determined optimal stimulation site (Avecillas-Chasin et al., 2015; Coenen et al., 2016) or to compare the imaging based targets to boundaries based on the evaluation of histological samples, which are typically accepted as ground truth for neurosurgical target volumes.

The purpose of our pilot study was to evaluate the technical feasibility of DTI based probabilistic tractography for localizing target areas that are commonly used during functional neurosurgery. Three targets were selected for this evaluation based either on their importance as a primary location targeted for the alleviation of the symptoms of movement disorders (Vim or VL_pv_ nucleus), or as a putative white matter target for ablative therapy (cerebello-thalamic or dentate-rubro-thalamic tract), and lastly, a possible “no-go” area to avoid adverse effects during deep brain stimulation or ablative therapy (VPL nucleus with somatosensory connections). We aimed to test the feasibility of tractography using the following criteria. First, to demonstrate the correspondence to the histologically defined anatomical structures for the targets that have digitized 3D atlas data (VL_pv_ and VPL nucleus of the thalamus) in common neuroimaging space. Secondly, since various aspects of reproducibility represent a predictor of the expected accuracy of targeting during neurosurgery, we set out to quantify the reproducibility of tractography from the observed variability in target position across subjects and the corresponding reproducibility within a subject but on different MRI scanners.

## Materials and Methods

### Study Population and Image Acquisition

Four volunteers with no neurological disorders participated in the study, and for each subject (ages: 28, 37, 40, and 52 years, gender: three males and one female) repeated scans were acquired on clinical MRI scanners of different manufacturers resulting in 10 different acquisitions. Diffusion weighted magnetic resonance imaging (DWI) sessions were carried out on three different 3.0T MRI systems: Siemens Skyra (Siemens Healthcare, Erlangen, Germany), GE Discovery MR750 (GE Healthcare, Milwaukee, WI, USA) and Phillips Ingenia (Phillips Healthcare, Best, The Netherlands), using spin echo echo-planar imaging sequences. The DWI acquisition details are summarized in **Table 1**. We will further refer to the scanners as Scanners 1, 2, and 3, not respecting the order of manufacturers in any depictions or tables. Reconstructed images were stored in DICOM format and were transferred to external workstations for image-processing. MRI scans were carried out with ethical approval and informed consent was obtained for all subjects.

**Table 1:** Diffusion tensor imaging acquisition parameters used in the study.

	Scanner 1	Scanner 2	Scanner 3
Number of diffusion weighting directions	32^∗^	32^∗^	32^∗^
Sequence type	2D EPI	2D EPI	2D EPI
B-factor	1000	1000	1000
Image matrix	128 ^∗^ 128,	112 ^∗^ 112,	96 ^∗^ 96,
	1.875 ^∗^	2 ^∗^ 2 mm	2 ^∗^ 2 mm
	1.875 mm		
Slice spacing	4 mm	2 mm	3.6 mm
TE/TR	95/4300 ms	92/10242 ms	80/6000 ms
NEX	1	1	1
Flip angle	90	90	90


*^∗^Protocol based on the study by Jones et al. (1999).*

### Image Processing

The following image processing steps were performed on the DWI data: (1) estimating symmetric tensors in each voxel of the diffusion data and using the tensor’s eigenvalues to calculate secondary to generate DTI data, parametric maps, such as the fractional anisotropy images, (2) spatial standardization of DWI data to a standard neuroimaging template space, (3) estimation of intra-voxel distribution of multiple fiber populations. We carried out the DTI processing steps with the FMRIB Diffusion Toolbox in the FSL software package (Jenkinson et al., 2012). Fractional anisotropy images (maps characterizing the directionality or “orderedness” of diffusion) were calculated using an established approach described elsewhere (Basser and Pierpaoli, 1996; Basser et al., 2000). We performed non-linear spatial standardization to enable inter-subject comparison of anatomy, and quantitative evaluations are reported in standard neuroanatomical space throughout the manuscript. For each subject, fractional anisotropy images were used to determine a deformation field which transforms the images to a common reference space, the FMRIB58 fractional anisotropy template (identical to MNI-152 space), by means of the FNIRT non-linear registration algorithm available in the FSL package. The characterization of fiber distributions was carried out using a Bayesian procedure (Behrens et al., 2003b) which searches for two fiber populations in each image voxel in such a way that the possible orientations of diffusion displacements best fit the observed DWI data.

### Mapping Connectivity-Based Targets within the Thalamus

Following the methods reported in the literature (Behrens et al., 2003a; Johansen-Berg et al., 2004, 2005; Klein et al., 2010; O’Muircheartaigh et al., 2011; Pouratian et al., 2011), thalamic connectivity-based subdivisions were defined by running probabilistic tractography from the thalamus volume to predefined cortical regions. Probabilistic fiber tracking was performed in DTI space to avoid interpolation of data; only the seed masks and the results were transferred to the standard space. The connection strength between each seed voxel and every remote brain voxel in the target regions was estimated as the probability of a tract reaching the target through a trajectory guided by the model of local diffusion characteristics. For each thalamic voxel, a counter variable increased when the emitted tracing samples entered any of the cortical masks, consequently resulting in back-projected connectivity probability maps corresponding to cortical territories delineated by using the Harvard-Oxford Atlas (Desikan et al., 2006). For each image voxel in standard space, 5000 tracing samples were used; loops and recursions were not allowed during fiber tracking. The images were divided by the total number of tracing particles that reached their destination (=“waytotal” count).

Based on post mortem neuroanatomical data and previous *in vivo* studies on the cortico-thalamic system (Kievit and Kuypers, 1977; Yeterian and Pandya, 1988; Behrens et al., 2003a; Gallay et al., 2008), we considered the following cortical targets to best determine the connectivity-based subdivisions within the lateral thalamus. For fiber tracking, it was necessary to define a seed and remote target region. Since the VL_pv_ or Vim nucleus is known to project to primary motor and premotor regions and also to the cerebellum (Hyam et al., 2012), the seed region was defined as the entire thalamus volume and the target regions were determined in two steps. First, the precentral gyrus in the Harvard-Oxford Atlas was selected as the target for mapping the thalamocortical connectivity of the primary motor cortex. Then, the targets were extended to include the interconnections of the VL_pv_ nucleus, such that the remote tractography targets included the entire cerebellar volume, supplementary motor cortex and primary motor cortex (precentral gyrus) in the relevant standard atlases (Desikan et al., 2006). The VPL_a_ and VPL_p_ nuclei are known to be connected to somatosensory cortical areas and therefore the remote target was defined by using the postcentral gyrus volume in the Harvard-Oxford Atlas.

### Anatomical Landmarks: Definition Using a Statistical Shape Model Based 3D Atlas of the Human Thalamus

Throughout the manuscript the nomenclature and anatomical definitions for the nuclei of the thalamus are based on the descriptions of Morel et al. (1997). Our study exploits the results of a recent study, which constructed a digitized 3D mean atlas using the histologically derived thalamic nucleus volumes by Morel et al. (1997), Niemann et al. (2000), and Krauth et al. (2010). Based on this atlas and the non-linear alignment described in a previous work (Jakab et al., 2012), high-resolution meshes of the thalamic nuclei were transformed to the standard MNI152 space. The individual nuclei volumes from the atlas were then re-sampled to an isotropic 1 mm image grid and image labels were constructed from the meshes. The transformation was based on a statistical shape model procedure (Blanc et al., 2009; Jakab et al., 2012), in which the predictor of intra-thalamic geometry was the thalamus outline, as derived from the manual segmentation of the thalamus in the T1-weighted image template in MNI space. While the original shape model atlas was constructed from seven histologically defined samples and hence incorporates population variability, for our study, the mean 3D volume of this dataset was used. The following structures were used from the atlas: VL_pv_: ventro-lateral nucleus posteroventral part (analogous to the Vim nucleus), VPL_p_: ventral-posterolateral nucleus posterior part and the whole-thalamus volume for visualization purposes. The MNI-transformed thalamus atlas and the atlas based targets used in our study are illustrated in **Supplementary Figure S1**. For illustration purposes, we used the histology based reconstructions provided by the Big Brain Atlas. This representation was based on the optically balanced non-linear matching of histological sections to a standard neuroimaging space (Amunts et al., 2013).

### Tracking the Cerebello-Thalamic Tract

Probabilistic fiber tracking by means of Probtrackx in the FSL software was used to map the CTT in the study population using identical tracking settings to those utilized for the thalamus connectivity maps. Our method was based on the description by Kwon et al. (2011) who reported initial results on the feasibility of DTI in reconstructing this fiber pathway. Using a cerebellar atlas in standard neuroimaging space (Diedrichsen et al., 2009), the seed region to initiate the tractography was the dentate nucleus contralateral to the thalamus, while the final target was the entire thalamus volume. Fibers were kept if they passed through the following way-point targets in the given order: first, the contralateral superior cerebellar peduncle and then the ipsilateral red nucleus region dilated by four voxels. Regions used for reconstructing the tract are depicted in **Supplementary Figure S2**.

### Quantitative Evaluations

Since the calculated connectivity-based targets (CBT) were extracted from probabilistic maps, their borders must be defined using arbitrary probability thresholds. The threshold choice will inherently affect the size of the target volume and its overlap percentage with the atlas based nuclei, e.g., a lower connectivity threshold will result in higher overlap between subjects, but the volume will be less specific to the targeted anatomical location. To achieve identical criteria for the threshold ranges across the study subjects, we used a range of percentile values of all non-zero voxel intensities to cut-off the connectivity values from the 50th to the 100th percentile, thereby selecting the voxels with the highest probability of connection to the cortical target regions. In practice, 50th percentile threshold meant that a large volume of the CBT is kept including regions of low probability of being interconnected to the relevant cortical region, while a 100th percentile threshold corresponded to the point which had the largest probability of connection to its target. All overlap and distance metrics were iteratively determined over this entire range of values. The dependency of the overlap with anatomical landmarks and the reproducibility was tested by using the following quantitative metrics:

1. Euclidian distance (mm) between the center-of-gravity (mid-point) point of the CBT and the relevant atlas targets. The coordinates for the center-of-gravity points of the thresholded connectivity maps and the atlas targets were calculated using the *fslstats* tool in the FSL software package.
2. Volumetric overlap between the CBT and atlas targets, determined by the Dice overlap (0–1), The Dice overlap coefficient D was defined as:

$$D=\frac{2|X\cap Y|}{|X|+\left|Y\right|}$$

where X and Y are the two volume sets to be compared.

The first metric assumes that we use point-like targets, either determined by connectivity or a 3D atlas model, the latter assumes that the targets consist of volumetric label maps relevant for surgical localization. Quantitative evaluations of the connectivity maps and 3D voxelised atlas volumes and the visualizations were carried out with Matlab R2010a for Windows (The Mathworks Inc., Nattick, MA, USA) using in-house developed algorithms.

## Results

### Correspondence of Thalamic Connectivity-Based Subdivisions to Atlas-Based Landmarks

The center-of-gravity points of the connectivity maps were located inside the atlas based targets in all cases and all scans. Center-of-gravity points are organized according to somatotopy along the lateral border of the thalamus, such that the connections to precentral gyrus are more anterior than the postcentral gyrus connections. In the present study, we described the correspondence of the VL_pv_ nucleus to two CBTs: the CBT using the precentral gyrus as target (“precentral connections”) and the CBT using a more extended *a priori* target list of the VL_pv_ nucleus (“VL_pv_ connections”).

**FIGURE 1:**
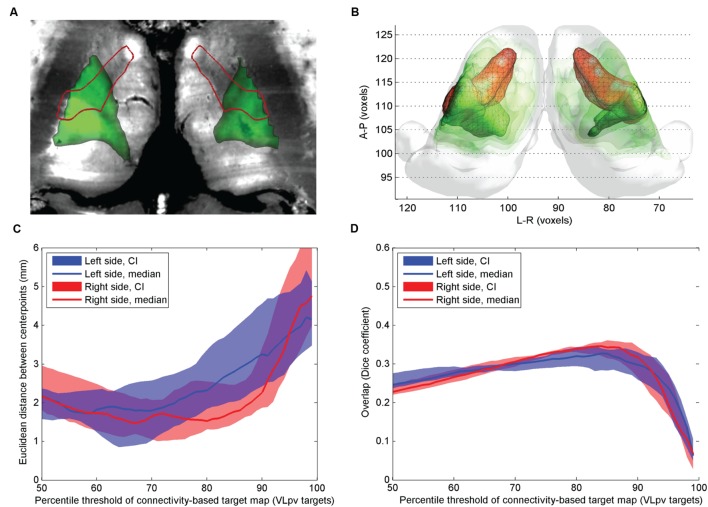
**Comparison of atlas and CBTs of the VL_pv_ nucleus.** **(A)** Cross-sectional image derived from histological sections, in standard space (Big Brain Atlas, Amunts et al., 2013) with VL_pv_ overlaid (red outlines) and the CBT (green color scale)**; (B)** 3D visualization of the CBT and atlas targets: connectivity-based targets were visualized in green color with different opacities reflecting to the summed probability across the study population (50–95th percentile range) while the VL_pv_ was depicted in red 3D mesh**; (C)** Euclidian distance of the center-of-gravity of the CBT to the atlas based target: dependency on the applied threshold values; **(D)** volumetric overlap with atlas based targets: dependency on the applied threshold.

**FIGURE 2:**
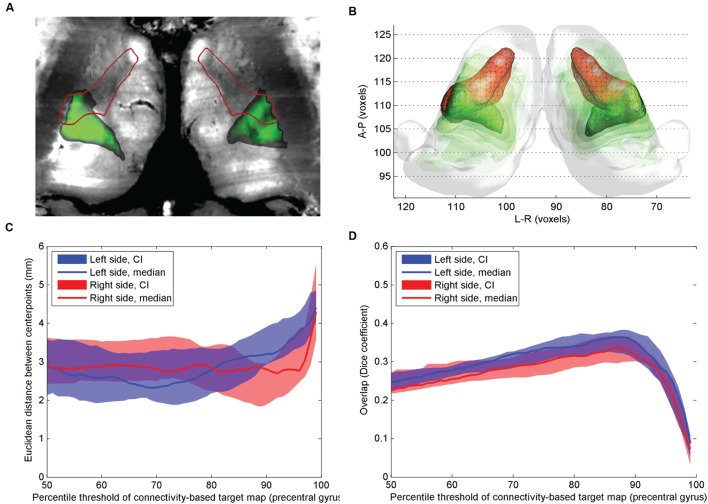
**Comparison of atlas based VL_pv_ nucleus and CBT using precentral gyrus connections.** **(A)** Cross-sectional image derived from histological sections, in standard space (Big Brain Atlas, Amunts et al., 2013) with VL_pv_ overlaid (red outlines) and the CBT from the precentral gyrus (green color scale)**; (B)** 3D visualization of the CBT and atlas targets: connectivity-based targets were visualized in green color with different opacities reflecting to the summed probability across the study population (50–95th percentile range) while the VL_pv_ was depicted in red 3D mesh**; (C)** Euclidian distance of the center-of-gravity of the CBT to the atlas based target: dependency on the applied threshold values; **(D)** volumetric overlap with atlas based targets: dependency on the applied threshold.

While there was a good agreement of the volumes along the antero-posterior axis of the most lateral parts of the thalamus, the connectivity patterns did not overlap with the medial parts of the VL_pv_ or VPL nucleus, and only partially overlap with more medially located thalamic nuclei with known motor connectivity, such as the centro-median (CM) nucleus. In the midline, the VL_pv_ was oriented more anteriorly, while the CBT defined this nucleus differently: the connectivity-based segment appeared wedge-shaped (**Figures 1** and **2**), with the tip oriented toward the center of the thalamus.

The mid-point distances between CBT and the anatomical target for the VL_pv_ nucleus were lowest on the population level when the connectivity maps were thresholded at the 65–70th percentile (left side) and between the 80th and 90th percentile (right side), and remained below 2.5 mm if the threshold was set to lower than the 85th percentile on the left side and 92nd percentile on the right side (**Figure 1D**). The VL_pv_ nucleus overlapped modestly with the CBT, demonstrating greater overlap in the lateral thalamus regions (**Figure 1C**). While the lateral segments were correctly matched visually to the VL_pv_ nucleus, the maximum value of the Dice coefficient was modest, median *D* = 0.32 (right side, threshold: 87th percentile) and median *D* = 0.30 (left side, threshold: 90th percentile). While the calculations were carried out in MNI152 standard and symmetrical template space, and the thalamic seed volumes were also symmetric, we noted an apparent left–right difference between the CBT volumes (**Figures 1A,B**). Regarding other nuclei that showed minimal overlap with the CBT of the VL_pv_: the CM nucleus showed no consistent overlap with the CBT volume (neither using VL_pv_, nor the precentral targets), and only moderate overlap was observed in the most medial regions of the CBTs.

The precentral connections also showed moderate overlap with the atlas based VL_pv_ volume (**Figure 2**): the CBT calculated using a single precentral target region overlapped slightly more with the atlas target compared to the CBT defined with additional (cerebellar, supplementary motor and primary motor) targets, and no interhemispheric asymmetry was observed (**Figure 2B**). Using a threshold at the 89th percentile (left side) and 88th percentile (right side), a median Dice overlap of 0.34 and 0.31 was measured on the left and right sides, respectively (**Figure 2C**). The pointwise distances from the atlas based targets were in the range of 2.2–3 mm in the connectivity range between the 70th and 86th percentiles (**Figure 2D**).

**FIGURE 3:**
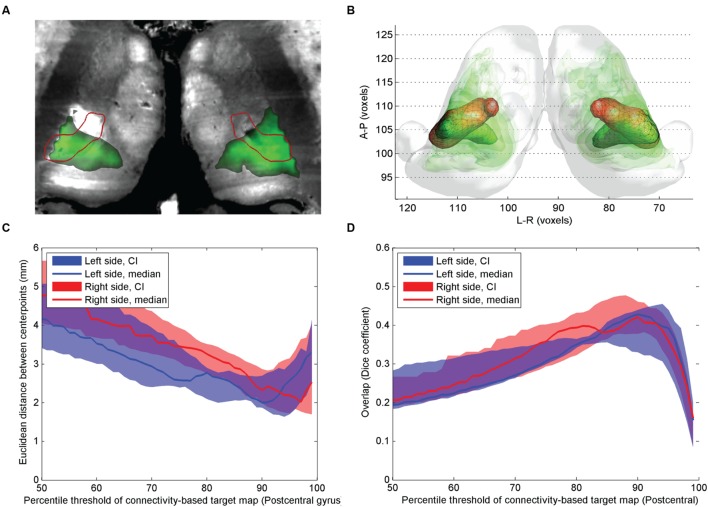
**Comparison of atlas based VPL_p_ nucleus and CBT using postcentral gyrus connections.** **(A)** Cross-sectional image derived from histological sections, in standard space (Big Brain Atlas, Amunts et al., 2013) with VPL_p_ overlaid (red outlines) and the CBT from the postcentral gyrus (green color scale)**; (B)** 3D visualization of the CBT and atlas targets: connectivity-based targets were visualized in green color with different opacities reflecting to the summed probability across the study population (50–95th percentile range) while the VPL_p_ was depicted in red 3D mesh**; (C)** Euclidian distance of the center-of-gravity of the CBT to the atlas based target: dependency on the applied threshold values; **(D)** volumetric overlap with atlas based targets: dependency on the applied threshold.

In comparison with the previous two targets, the overlap between the postcentral gyrus connections and the atlas based VPL_p_ (and VPL_a_) was larger (**Figure 3**). The mid-point distances between CBT and anatomical target for the VPL nucleus were lowest on the population level when the connectivity maps were thresholded at the 90th percentile, with median distances of 2.1 mm for the left and 2.4 mm for the right side (**Figure 3C**). The volumetric overlap between the CBT and the ABT for the VPL nucleus was generally higher than in the other examined thalamic nuclei. Consistent with results from the other nuclei, the maximum overlap was achieved by thresholding around the 90^th^ percentile of the connectivity values (**Figure 3D**).

### Selecting the Optimal Connectivity Threshold for Connectivity-Based Targets

**FIGURE 4:**
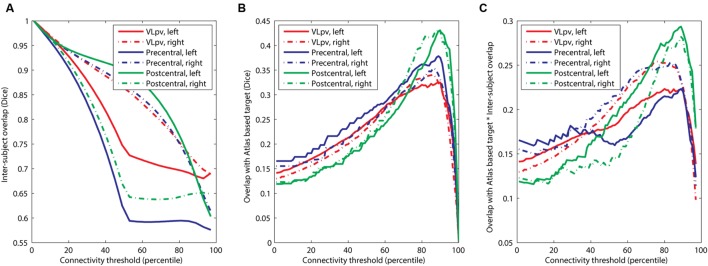
**Optimization of the connectivity threshold applied to connectivity based intrathalamic targets.** **(A)** Inter-subject overlap as a function of the applied connectivity threshold**; (B)** overlap with anatomical atlas as a function of the applied connectivity threshold**; (C)** determining the population-level optimal threshold value as a maximum point of the function.

Our results suggest that while the agreement with the atlas targets is higher if the connectivity threshold is increased (for example, a maximum is seen in **Figure 3C** at the 90th percentile threshold), the inter-subject reproducibility was decreased at larger connectivity thresholds. Therefore, it was necessary to determine a consensual cut-off value which represents a trade-off between the two feasibility metrics. First, we investigated the effect of connectivity probability thresholding on the expected reproducibility of CBT maps. The lower the connectivity threshold, the higher the inter-subject overlap (**Figure 4A**). This means, however, that the CBTs become less specific for the given neurosurgical target: as the connectivity threshold values converge to zero, almost the entire thalamus is included as a potential target volume, which is anatomically incorrect. In order to find the optimal connectivity-based threshold, representing a compromise between larger inter-subject reproducibility and larger overlap with the atlas target, we multiplied the two overlap metrics and located the maximum value of this combined metric (**Figure 4**). To provide a consensual value, we multiplied the median values of population-overlaps and the median values of the correspondence to the anatomical atlas. In a similar manner as for the overlap with the anatomical target (**Figure 4B**), the consensus metric also had a maximum, which was used to determine the optimal cut-off value (**Figure 4C**).

The optimal connectivity threshold values were the following. VL_pv_ left: 80th percentile; VL_pv_ right: 76th percentile; precentral CBT left: 90th percentile; precentral CBT right: 84th percentile; postcentral CBT left: 89th percentile; postcentral CBT right: 89th percentile. Throughout the study, these optimized cut-off values are used for visualization and further evaluation of reproducibility.

### Reconstruction of the Cerebello-Thalamic Tract: Tractography-Based Anatomy

**FIGURE 5:**
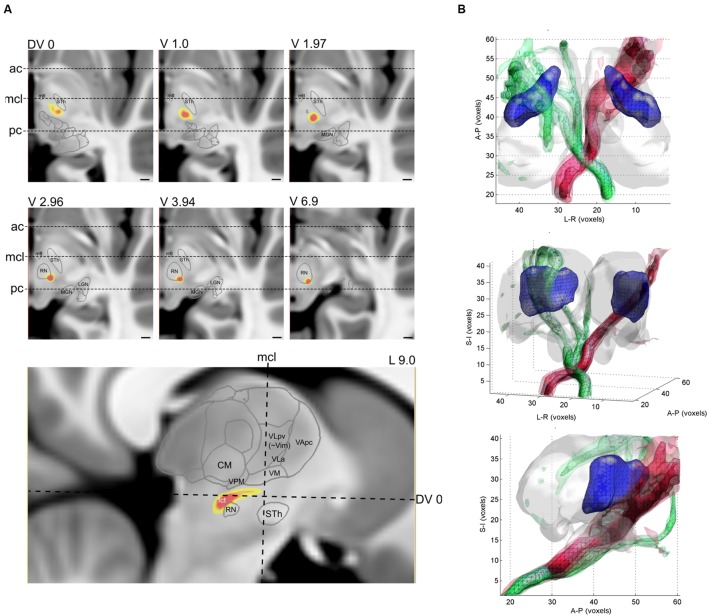
**Reconstruction of the CTT with diffusion tractography.** **(A)** population-averaged connectivity map (yellow-red color) overlaid onto a cross-sectional image (T1-weighted MRI). Slice levels are relative to the AC-PC plane, and three major anatomical landmarks have been annotated, ac, anterior commissure, mcl, mid-commissural line, pc, posterior commissure. **(B)** 3D visualization of the population-averaged CTT. Different opacities correspond to the population probability from 50th percentile to 90th percentile.

The CTT was reconstructed for each of the 10 datasets of the four participants. The typical trajectory of the DTI based reconstruction was the following. The tract emerged from the ipsilateral dentate nucleus, crossed to the opposite hemisphere at the level of superior cerebellar peduncle, and then passes medially and across the red nucleus. Cross-sectional images with annotations of the thalamic nuclei and major anatomical landmarks are shown in **Figure 5A**. The intra-thalamic terminations of the CTT overlapped with the VL_pv_ nucleus, but due to the technical limitations of DTI tractography, the CTT fibers appeared outside the thalamus and subthalamus, and continued to a diverse set of supratentorial areas. In order to provide plausible visualizations of the tract, we limited the field of view to the subthalamus and thalamus. A notable left–right asymmetry was detected on the population level, in which the right CTT (originating from left cerebellum) was found to be more strongly connected to its pathway targets. Compared to the intra-thalamic targets, the CTT reconstruction showed significantly larger variability across the population. In some cases, the pathway was split and alternative routes were taken, possibly due to errors in the estimation of crossing fibers. In some cases, zero or a very low inter-subject overlap was reported using the 95th percentile of connectivity values, possibly due to splitting of the pathway into alternative routes, or due to the diminished sensitivity of locating the fibers at a single connectivity threshold. As observed for the intra-thalamic CBTs, the inter-scanner variability was similar to the inter-subject variability (**Table 2**).

**Table 2:** Reproducibility measures of the CBTs: inter-subject and inter-scanner variability.

Thalamic nucleus, CBT	Within-subject, inter-scanner overlap (Dice, mean ±*SD*)	Inter-subject, within-scanner overlap (Dice, mean ±*SD*)	Difference between inter-scanner and inter-subject variability (*p*-value)	Overall overlap (Dice, mean ±*SD*)
Left VL_pv_-proper	0.651 ± 0.112	0.720 ± 0.123	*p* = 0.195	0.662 ± 0.129
Right VL_pv_-proper	0.703 ± 0.065	0.712 ± 0.095	*p* = 0.806	0693 ± 0.084
Left VL_pv_-precentral	0.567 ± 0.141	0.631 ± 0.069	*p* = 0.235	0.580 ± 0.119
Right VL_pv_-precentral	0.603 ± 0.082	0.651 ± 0.087	*p* = 0.209	0.587 ± 0.121
Left VPL_p_-postcentral	0.745 ± 0.059	0.583 ± 0.159	*p* = 0.0166^∗^	0.637 ± 0.136
Right VPL_p_-postcentral	0.429 ± 0.103	0.331 ± 0.09	*p* = 0.0325^∗^	0.641 ± 0.109
Left CTT	0.263 ± 0.369	0.213 ± 0.223	*p* = 0.728	0.239 ± 0.253
Right CTT	0.240 ± 0.364	0.328 ± 0.358	*p* = 0.586	0.261 ± 0.296


*^∗^Significant result, *p* ≤ 0.05.*

### Reproducibility of Connectivity-Based Targets

**FIGURE 6:**
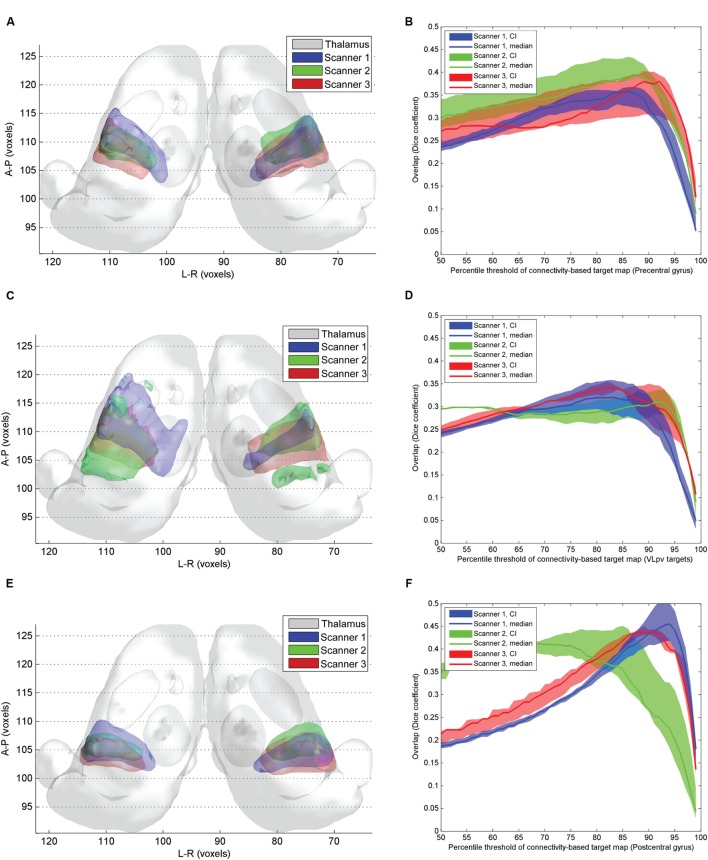
**Inter-scanner reproducibility of connectivity based intrathalamic targets.** **(A)** 3D visualization of the inter-scanner variability of precentral CBT over the study population; **(C)** 3D visualization of the inter-scanner variability of VLpv CBT over the study population; **(E)** 3D visualization of the inter-scanner variability of VPLp CBT over the study population; **(B)** dependency of the inter-scanner variability of the precentral CBT volume on the applied connectivity threshold; **(D)** dependency of the inter-scanner variability of the VLpv CBT volume on the applied connectivity threshold; **(F)** dependency of the inter-scanner variability of the VPLp CBT volume on the applied connectivity threshold.

While the pre- and postcentral gyrus CBT were largely reproducible across different scanners (**Figures 6A,B,E,F**), the VL_pv_ CBT showed considerable variability (**Figures 6C,D**), as well as a larger volume and a modest interhemispheric asymmetry. We investigated the inter-scanner reproducibility of the CBT structures by comparing both the volumetric overlaps and the center-point distances. The maximum of the volumetric overlap was observed at similar connectivity percentiles as in the previous experiments; however, the center-point distances measured showed considerable differences between scanners of different vendors. The results obtained from Scanner 1 were found to match better the center-points of the ABTs, and the minimum Euclidean distances were below 2 mm when the CBT was thresholded at the 75th percentile. A similar effect was not observed using the data from the other two scanners. The volumetric overlaps for each of the 3 CBTs showed similar curves with maximum peaks at 90–95th percentiles. As summarized in **Table 2**, the overlap between CBTs across all subjects and scans was between 58 and 69% for the thalamic targets. When decoupling the variability into inter-subject within-scanner and intra-subject inter-scanner variability, we observed that for most of the structures, the inter-subject overlap was similar to the inter-scanner variability. Only in the case of the postcentral gyrus connections were the inter-scanner variability significantly higher (**Table 2**), meaning that the vendor type (Scanners 1–3) caused more variability than the anatomical differences between individuals after non-linear standardization.

While the thalamic CBTs were defined by setting a cut-off value of connection probability based on the optimization step described above, we were not able to perform this optimization step for the CTT due to its specific shape and configuration. Since the CTT showed less variability in the sub-thalamic region, we decided to calculate the variability measures after restricting the volume of the tract to a 40 mm subvolume symmetrically around the AC-PC plane, which is most commonly used for neurosurgical targeting (20 mm ventral and 20 mm dorsal). After limiting to this volume and setting the connectivity value to the top 90th percentile of connections, a low overlap was observed for the CTT (**Table 2**). The CTT was significantly more reproducible across subjects on the right side (*D* = 37.4%) than on the left (20.7%), and the left CTT showed a similar phenomenon to the intrathalamic targets in that the inter-scanner variability exceeded the inter-subject variability.

## Discussion

The use of DTI based tractography of thalamocortical connections and the CTT for visualization of neurosurgical targets must be preceded by validation efforts that evaluate the technical feasibility in the clinical setting. By reconstructing various subdivisions of the thalamus in volunteers, we were able to quantify the overlap and pointwise distances to atlas-based anatomical targets. A particularly challenging aspect is to capture the population variability and scanner dependency of such reconstructions, and to the best of our knowledge, no previous study has tested the scanner dependency of these neurosurgically relevant thalamic and subthalamic structures. During this step, we optimized threshold values that delineated the connectivity based intrathalamic maps to obtain a volume: while the population variability was lower when lower thresholds were applied, the correspondence to the anatomical target was higher when applying higher connection thresholds. This effect appeared to have a maximum and therefore an optimal connection threshold could be determined. The same procedure was not possible for the CTT, as we do not have digitized 3D atlas data for this structure.

### Functional Neurosurgical Targets in the Thalamus: Feasibility of the Tractography Based Approach

For reconstructing the fiber pathways that are interconnected with the thalamus, we chose to use probabilistic diffusion tractography. The first reports have confirmed that this technique is capable of resolving the complex within-voxel orientations of fibers within the gray-matter rich thalamus (Behrens et al., 2003a; Johansen-Berg et al., 2005), and that probabilistic tractography yields major advantages over deterministic approaches despite the much higher computational load (Descoteaux et al., 2009). For example, when estimating the orientation density function (i.e., the distribution of possible fiber direction within an image pixel), the deterministic approach only takes the most probable diffusion orientation, while the probabilistic framework models more complex fiber geometries, such as fanning, crossing or splaying. On the other hand, probabilistic tractography suffers from connectivity values that decrease with the distance from the initial seed point due to the fanning of fiber bundles (Descoteaux et al., 2009).

The reported overlap of the atlas volumes and the CBTs inside the thalamus was consistently lower than 50%, which can be explained by the known anatomical structure of the thalamus: while the lateral, especially ventrolateral parts of the thalamus have point-to-point interconnections with the cortex, the more medial zones have diffuse connectivity, such as the CM or centrolateral nucleus (Kievit and Kuypers, 1977; Morel et al., 1997). It is likely that diffusion based techniques are only able to resolve the anisotropically organized axons within the thalamus, which do not form well circumscribed subdomains, but rather show a gradient of connections on the antero-posterior axis. These anisotropic structures mix with the afferent fibers of the thalamus, which are located more ventrally. This error presumably also stemmed from the fact that diffusion tractography cannot distinguish between afferent and efferent connectivity and hence the projections of the thalamus in the two directions (i.e., the cortex and spinal cord) cannot be separated. Compared to the connectivity based volumes, the VL_pv_ nucleus appears larger and extends more dorsomedially in the reconstruction in **Figures 1** and **2**. However, it has been shown by microelectrode recordings in tremor that the associated rhythmic burst was found in a restricted area of the ventrointermedius nucleus, and only the lateral and ventrocaudal part of each nucleus, which was defined as the kinesthetic zone, was involved (Ohye et al., 1993).

Our results indicate that the Euclidian distance to the centerpoint of the atlas volume can be kept below 2.5 mm by applying optimized threshold values. This distance was considerably larger than the reported distances to experimentally defined target locations during surgery; for example, Pouratian et al. (2011) found DTI derived connections to be 0.36 mm away from the optimal stimulation sites. It is likely, however, that the centerpoints of the atlas based targets do not represent the optimal stimulation sites and therefore our results may underestimate the accuracy obtained with tractography. The localization of effective tremor treatment sites, therefore, might be better reflected in the thalamocortical connectivity patterns than the exact borders of the cytoarchitecturally determined areas. This is also supported by studies that compared the adjacency of the target site to reconstructed fibers (Owen et al., 2008; Coenen et al., 2011b; Hunsche et al., 2013; Torres et al., 2014), and by studies that evaluated the network emerging from the stimulation sites (Klein et al., 2012).

In most previous studies, the CBTs were defined using a single target region of interest within the cortex, although there is experimental support from imaging studies that the Vim or VL_pv_ nucleus has dominant connections beyond those of the primary motor cortex. For example, Hyam et al. (2012) showed that the Vim has high connection densities to the cerebellum, as well as to the premotor and dorsolateral prefrontal cortex. This observation motivated us to use a more extended list of targets when back-projecting the connections into the thalamus: namely, in addition to the precentral gyrus, the cerebellum and premotor region were added. We observed that these additional target regions did decrease the center-point distance to the atlas-based target (**Figure 1**), but did not increase the overall reproducibility of the CBT. Furthermore, we observed interhemispheric asymmetry in the VL_pv_ CBT reconstruction, which was not the case when using only the precentral gyrus as remote connection target.

While a general good inter-patient reproducibility was found for the thalamic connectivity-based segmentations (Traynor et al., 2010; O’Muircheartaigh et al., 2011), especially for the lateral thalamus, little information is known about the dependency of scanner and sequence parameters. Interestingly, our study revealed that small differences in sequence settings and vendor-specific, underlying MR technology can contribute significant variability to the observed connectivity structure. In fact, the variability caused by scanner differences appeared to be similar or larger than the inter-subject variability within one scanner (precentral connections: 57–60% overlap between repeated scans on different scanners, 63-65% overlap between subjects on the same scanners, see **Table 2**). The non-linear matching used to normalize images to MNI space should reduce the inter-subject variability, but the uncertainty of the registration algorithm cannot be separated easily from the other apparent sources of variability, and significant inter-scanner variability remained even after normalization. The exact source of this inter-scanner variability is unknown, but may arise from a number of different hardware and software factors. While some vendors use a spectrospatial (water excitation) pulse for fat suppression, others use fat saturation methods, resulting in different limitations on the minimum slice thickness allowed. Differences in these excitation pulses, coupled with differences in gradient design, geometric distortion, and the use of wide vs. narrow bore systems may contribute to the observed inter-scanner variability, but future studies would be needed to elucidate further the sources of this observed variability. This inter-scanner variability further underscores the sensitivity of DTI and connectivity derived measures to sequence parameters and hardware parameters (Pfefferbaum et al., 2003; Takao et al., 2011, 2012; Bonilha et al., 2015; Kuhn et al., 2015).

### Targeting the Cerebello-Thalamic Tract Using Tractography

Using the seed and target list known from the literature (Kwon et al., 2011), we were able to reconstruct the 3D trajectory of the CTT (**Figure 5**) using current standard clinical DTI protocols. The trajectory is similar to what we know about the anatomy of this tract, especially in the subthalamic region: after crossing to the contralateral hemisphere, the tract projects into the thalamus by passing near the red nucleus and sending collaterals to it, and the tract is generally located posterior to the mid-commissural line around the AC-PC plane (Gallay et al., 2008; Kwon et al., 2011). This observation is in line with observations from studies that aimed to reconstruct the dentate-rubro-thalamic connections in clinical datasets. We were unable to compare the CTT volume to atlas derived data, and to the best of our knowledge, no such studies have yet been performed, although many authors aimed to correlate the effective treatment site location to the reconstructed DTI-based target. For example, Coenen and colleagues showed the feasibility of the CTT mapping in tremor alleviation in case reports (Coenen et al., 2011a,b), and in a group of 11 patients (Coenen et al., 2014). In their study, effective contacts were located in proximity to the DRT; in moderate tremor reduction, the target fields were centered on its anterior border while in good and excellent tremor reduction, the target fields focused on its center. While Schlaier et al. (2015) were able to reconstruct the CTT in 90% of their subjects, they concluded that contacts closer to the tract did not provide better clinical effects than distant contacts. In our study, a low reproducibility was found for the tract, even after limiting the field of interest to a limited supero-inferior range near the AC-PC plane. This further highlights the challenge of using routine clinical DTI scans to reconstruct this tract, and indicates that further tests of reproducibility and sequence dependency are needed. We assume that by using high angular imaging, the complex white matter anatomy of the subthalamic region can better be reconstructed.

### Study Limitations

Our study is limited by the small number of volunteers for whom we were able to acquire repeated DTI scans. This particularly limits our capability to test inter-subject variability and to decouple the variability caused by anatomical differences from the repeatability of imaging, uncertainty of fiber tracking and errors of image co-registration.

A major limitation in our study is the lack of ground truth information on thalamic fibers and the lack of population-wide histology-based atlases of white matter structures. This limits the generalisability of our validation: although we used a shape model that incorporated variability (Krauth et al., 2010; Jakab et al., 2012), it remains questionable if the mean volume in standard neuroimaging space is acceptable as a reference. It is particularly challenging to relate the diffusion tractography based structures to histological atlas. To achieve this goal, pre- or post mortem diffusion imaging with feasible image quality is needed, ideally with manual delineation of stained sections and a histological work-up identical to those in the classical atlases. Therefore, despite the technical feasibility of the tractography method, the present results do not encourage any changes to current practice in functional neurosurgery, rather they indicate that more comparative studies between MR and gold-standard anatomical or histopathalogical imaging are required.

While our study aimed to evaluate clinically feasible imaging protocols, such as the widely used 32-direction DTI, we assume that higher angular resolution diffusion datasets will reduce the variability associated with imaging, and will also allow us to resolve the challenging fiber anatomy of the thalamic and subthalamic region more accurately. For this, more sophisticated diffusion models and the resolution of multiple crossing fiber populations are necessary (Behrens et al., 2007).

Our study is further limited by its exploratory nature, and specifically the small participant group size. The widespread usage of probabilistic diffusion tractography for defining intra-thalamic and cerebello-thalamic targets must be preceded by large, preferably multi-centric validation efforts that test the technique during interventions and compare the results to those of the standard target localization methods. Furthermore, the inter-surgeon variability should also be evaluated, which can be a critical point when applying localization approaches that rely heavily on image processing with complex parameter settings.

## Conclusion

In this pilot study, we demonstrated the reproducibility of DTI based tractography to map targets with relevance to preoperative planning. The accuracy with regard to the center-points of atlas-based targets was around 2.5 mm, and a relatively low volumetric overlap of 35–40% was observed. We conclude that the connectivity based subdivisions can only predict the lateral and ventral segments of the Vim or VL_pv_ nucleus and the postcentral connections emerging from the VPL nucleus. The applicability of connectivity-based segmentation methods with the current clinical sequence parameters is further limited by large scanner-to-scanner variability of the CBTs. Further investigations using higher angular resolution DTI are needed to elucidate the sources of this variability and to assess the precise contribution of subject-to-subject anatomical variability.

## Author Contribution

AJ, BW, EM, JT, TY, GS, and RT designed the study. AJ has written the manuscript, performed the analysis and created figures. KK has performed the analysis and contributed to the manuscript. RT has acquired data and contributed to the manuscript. EM has written the manuscript and provided expert consultation. MP has acquired data and provided expert consultation. All authors have read and edited the manuscript.

## Conflict of Interest Statement

The authors declare that the research was conducted in the absence of any commercial or financial relationships that could be construed as a potential conflict of interest.

## Supplementary Material

**FIGURE S1 | The statistical shape model based atlas of the human thalamus used in our study.**

**FIGURE S2 | Reconstruction of the CTT using diffusion tractography.** 3D visualization of the seed and waypoint target regions used during fiber tracking.

## Abbreviations

AC: anterior commissure
CBT: connectivity based target
CTT: cerebello-thalamic tract
DTI: diffusion tensor imaging
DWI: diffusion weighted imaging
MNI: Montreal Neurological Institute
MRI: magnetic resonance imaging
PC: posterior commissure
Vim: nucleus ventralisintermedius
VL_pv_: nucleus ventrolateralis part posteroventralis
VPL_p_: nucleus ventralisposterolateralis posterior part
