# Supplementary figures and images for: Feasibility of Diffusion Tractography for the Reconstruction of Intra-Thalamic and Cerebello-Thalamic Targets for Functional Neurosurgery: A Multi-Vendor Pilot Study in Four Subjects
Source: Front Neuroanat. 2016 Jul 12;10:76. doi: 10.3389/fnana.2016.00076 (PMC4940380; doi:10.3389/fnana.2016.00076)

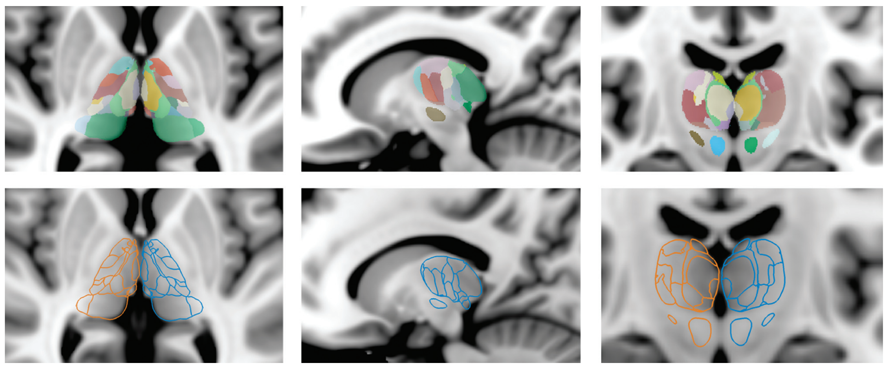

Supplement: Supplementary file 1 [file Image_1.TIF]

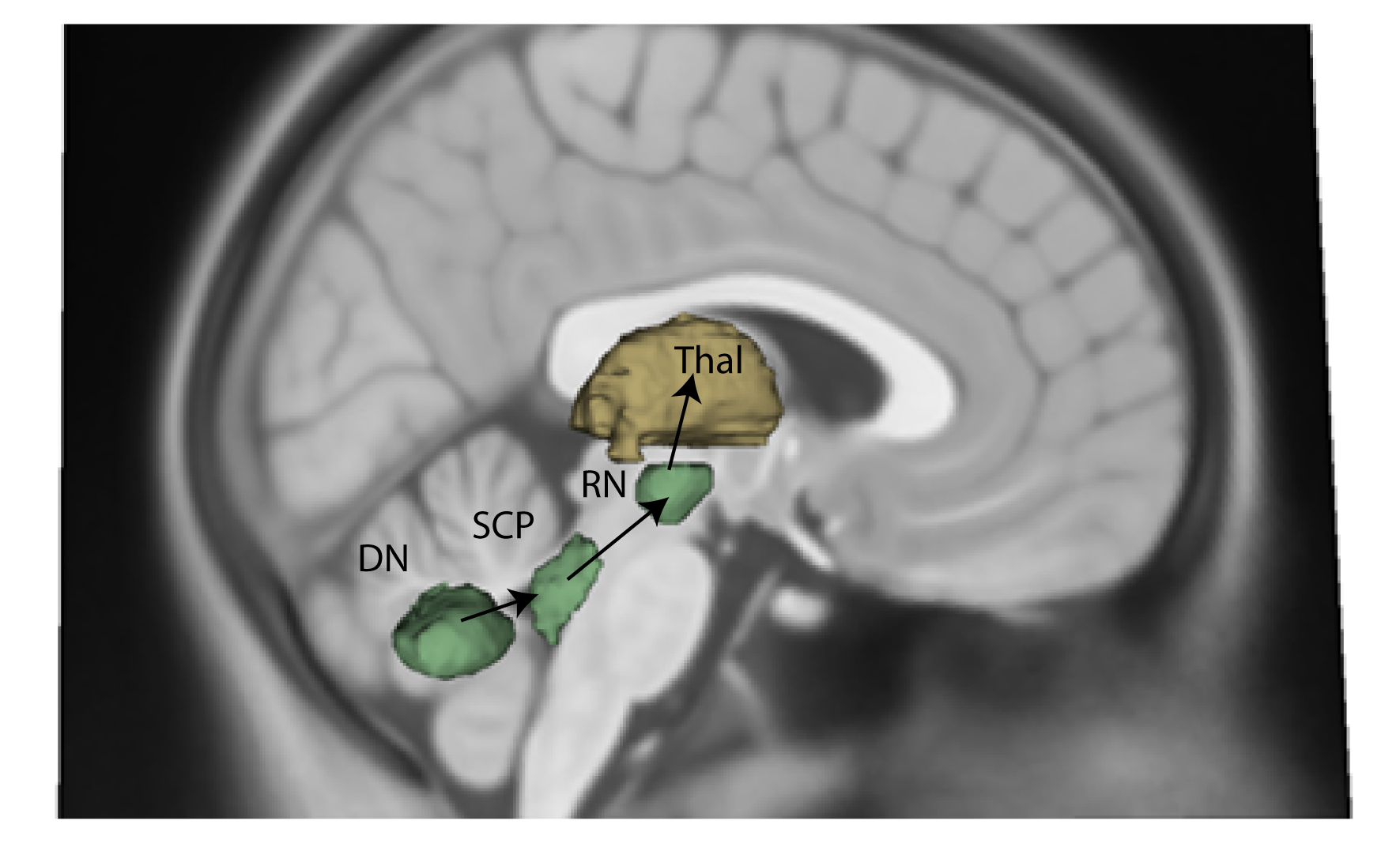

Supplement: Supplementary file 2 [file Image_2.TIF]
